# Legal Notes for Institutional Workers

**Published:** 1914-05-16

**Authors:** 


### LEGAL NOTES FOR INSTITUTIONAL WORKERS.

### A Mental Specialist's Fees.

The point in *Hamilton* v. *Bryant*, 30 T. L. R. 78, was a simple and practical one. By written agreement the defendant had agreed to pay to the plaintiff, who was a mental specialist, £500 a year for the care and maintenance of her daughter, payment to be made once a quarter. On April 7, 1913, the defendant gave plaintiff three months' notice terminating the agreement. The agreement was dated May 15, 1899, and the argument of the plaintiff was that it could only be terminated at the end of a year—*i.e.*, on May 14 in any year—by reasonable notice previously given. According to this contention the agreement would have subsisted for nearly a year beyond the date at which the defendant desired to terminate it. The Court, however, held that it was an implied condition of such an agreement that it could be terminated by reasonable notice given at any time, and with this direction before them the jury found that the defendant had given reasonable notice, and accordingly the plaintiff's claim for breach of agreement was dismissed. Mr. Justice Atkin, in charging the jury, said that the agreement was subject to the ordinary rule that where a contract was entered into for a certain object, conditions which must have been in the minds of both parties as necessary to carry out that object could be implied in the contract. Here the defendant's daughter was handed over to the plaintiff as an experienced doctor who was to give her care and attention. It appeared to him obvious that circumstances might arise in which it might be desirable that the patient should be removed at a comparatively short notice and not forced to remain in the plaintiff's house, it might be, nearly a year longer than was desirable. On the other hand, it might be desirable from the plaintiff's point of view, bearing in mind the claims of his other patients, that he should be able to procure her removal on a similar notice, as he might not be able to give her proper attention. He thought, therefore, that, in the circumstances, a right to give reasonable notice of termination was implied in the agreement.

### Education Authorities and Medical Associations.

The well-known and leading case of *Hillyer* v. *St. Bartholomew's Hospital*, 1909, 2 K. B. 820, has again been followed, and the principle of it in fact extended in *Davis* v. *London County Council*, 30 T. L. R. 275, where it has been laid down that, if an education authority under the Education Acts enter into an agreement with a medical association in regard to the performance of operations on school children, the education authority are not liable for the negligence of the persons performing the operation, provided they engage competent professional persons to perform it. The jury found that the anæsthetist concerned in the operation complained of had not been guilty of any negligence; and Mr. Justice Lush, in giving judgment for defendants with costs, said that in his opinion a contrary verdict would have imposed no liability on the defendants, as they had discharged their duty towards the plaintiff when they had employed competent professional men.

### Bequest to an Attendant.

The judgment of Mr. Justice Eve in *Re Lawson*, 30 T. L. R. 335, is decidedly questionable. The point before him was the interpretation of a clause, very common in wills, whereby a testator bequeathed one year's wages to each of his domestic servants who should have been in his service two years before his death, and should not be under notice to leave. It happened that the testator had been attended by a certificated male nurse, who had looked after him throughout the two years preceding his death, with the exception of four months, during which he (the nurse) was absent on holiday and received no wages. The defendant got his meals in the testator's house, but did not sleep there. It was held that the nurse was a domestic servant, that he satisfied the conditions of the will, and was entitled to the bequest.

### A Point for Public Health Officers.

An important question relating to public health administration and also one of some medical interest was decided in *Attorney-General* v. *Shoreditch Corporation*, 30 T. L. R. 382. By Section 5 of the Baths and Washhouses Act of 1878 a borough council may in winter close their public swimming-bath and allow it to be used for purposes of healthful recreation or exercise. It has been held in the case mentioned that the letting of the bath for a cinematograph entertainment was a letting for a "healthful recreation" within the meaning of the above provision.

